# Geospatial Analysis of Lumpy Skin Disease Outbreaks among Cattle in Uttar Pradesh, India, 2021–2022

**DOI:** 10.3390/pathogens13080611

**Published:** 2024-07-24

**Authors:** Isha Agrawal, Barkha Sharma, Ajay Pratap Singh, Csaba Varga

**Affiliations:** 1 Department of Pathobiology, College of Veterinary Medicine, University of Illinois Urbana-Champaign, Urbana, IL 61802, USA; ishaa3@illinois.edu; 2 Department of Veterinary Epidemiology, College of Veterinary Science and Animal Husbandry, U.P. Pandit Deen Dayal Upadhyay Pashu Chikitsa Vigyan Vishwavidyalaya Evam Go Anusandhan Sansthan, Mathura 281001, India; barkha16vet@gmail.com; 3 Department of Microbiology, College of Veterinary Science and Animal Husbandry, U.P. Pandit Deen Dayal Upadhyay Pashu Chikitsa Vigyan Vishwavidyalaya Evam Go Anusandhan Sansthan, Mathura 281001, India; drajay_vet@yahoo.co.in

**Keywords:** lumpy skin disease, India, spatial analysis, clustering, climate, cattle

## Abstract

The emergence of lumpy skin disease (LSD) among cattle in India is concerning. District-level data on LSD cases in Uttar Pradesh between 2021 and 2022 were analyzed. A stepwise spatial analytical approach was followed by first constructing yearly and monthly disease maps for LSD incidence rates (IRs), then spatially interpolating the LSD IRs, followed by evaluating the global and local clustering of LSD IRs and finally conducting spatial regression modeling. Overall, 5784 LSD cases from 6 districts and 112,226 cases from 33 districts were detected in 2021 and 2022, respectively. In the incremental spatial autocorrelation analysis, the highest global clustering of LSD IRs for the 2022 outbreak was detected at 196.49 km. For the 2021 LSD outbreak, one district with high-low and nine districts with low-high LSD IRs were identified in the eastern region of the state. For the 2022 LSD outbreak, 13 districts with high-high and 7 districts with low-high LSD IRs were identified in the western part of the state. A geographically weighted regression model identified the impact of climate (temperature and humidity) and land cover (pasture, fallow, and non-agricultural land) on LSD IRs. The study results can aid animal health authorities in developing LSD prevention and control programs.

## 1. Introduction

Lumpy skin disease (LSD), caused by a virus of the Capripox genus, is an emerging transboundary, notifiable disease primarily affecting large ruminants (cattle, water buffaloes, and other bovines) [1]. The virus is host-specific, causing disease in large ruminants of all ages [1]. The virus exhibits high morbidity and low to moderate mortality, varying with population susceptibility and virus strain [2,3,4]. The clinical signs of LSD include fever, enlarged lymph nodes, anorexia, depression, emaciation, and the development of skin nodules [5,6]. LSD transmission has been categorized into short and long transmission routes. The short transmission route includes direct contact between infected and susceptible animals and vector-borne transmission via blood-sucking arthropod vectors such as mosquitoes, biting flies, midges, and ticks [7,8]. The long transmission route involves the transportation of infected animals and windborne or vehicle-aided transport of vectors [8,9]. In addition, unregulated and illegal animal movement across state and international borders plays a critical role in transboundary disease introduction and spread into naïve cattle herds [9].

LSD cases first emerged in Asia in mid-2019 in Bangladesh [10], causing a transboundary spread of the disease. LSD outbreaks have been described in multiple Asian countries, including China, India, Nepal, Myanmar, Pakistan, Thailand, and Vietnam [10,11]. In India, the first LSD case was reported in August 2019 from the Mayurbhanj district of Odisha, a state close to the Bangladesh border [12]. Since its first detection, LSD spread to over 15 states by 2022, affecting over two million cattle and causing over 155,000 deaths in 2022 [4,12]. Uttar Pradesh, which ranks second in cattle production in India and raises around 18.8 million cattle [13], was among the affected states. The spread of LSD among cattle populations in India has caused economic losses through increased mortality; reduced milk production; and added disease prevention, management, and treatment expenses [14,15].

The cattle industry in Indian states, including Uttar Pradesh, is unorganized, with mostly small-scale cattle farms [16]. These farms have insufficient biosecurity and limited resources, facilitating disease spread within and between farms [16,17]. Moreover, unstructured and unregulated trade and transportation of cattle might promote disease spread within and across state borders [9,12]. In these settings, disease control efforts must be focused on strategically localized vaccinations and early restriction of animal movements [10]. However, efficient implementation of these measures requires up-to-date knowledge of the geographical distribution and spread of LSD among cattle populations.

Our previous study evaluated the spatial–temporal clustering and climatic risk factors of LSD in cattle populations during the 2022 outbreak in Uttar Pradesh [18]. However, additional analysis is needed to describe the monthly geospatial distribution and clustering of LSD incidence rates in Uttar Pradesh during 2021 and 2022 and to compare the two LSD outbreaks. This study intends to aid animal health authorities in developing focused disease prevention and control methods to allocate resources efficiently and set up early warning systems.

## 2. Materials and Methods

### 2.1. Study Region

**Figure 1:**
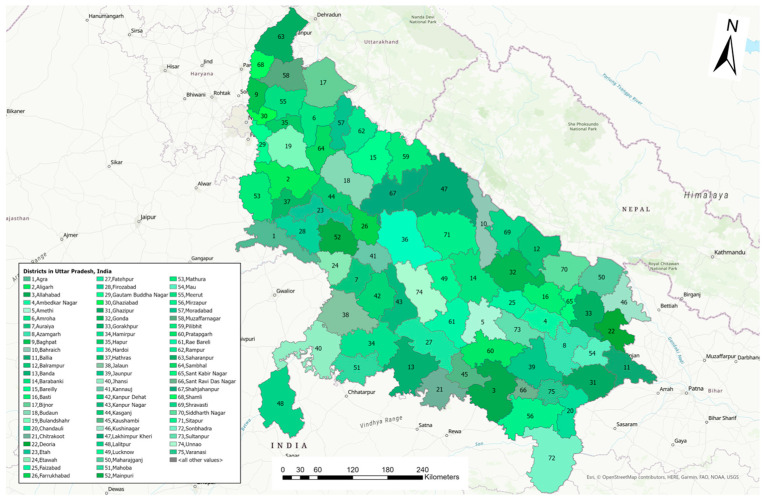
Map illustrating the state of Uttar Pradesh, India, and its districts (*n* = 75).

This study evaluated the 2021 and 2022 LSD outbreaks among cattle in Uttar Pradesh, India. Uttar Pradesh is a North Indian state (26.85° N 80.91° E) sharing borders with Nepal, a Union Territory (Delhi), and eight other Indian states (Rajasthan, Haryana, Himachal Pradesh, Uttarakhand, Bihar, Madhya Pradesh, Jharkhand, and Chhattisgarh). Uttar Pradesh has a land area of 93,023 square miles, encompassing 75 districts (Figure 1). The state is a leading dairy cattle producer, with a cattle population of 18.8 million [13].

### 2.2. Data Collection

#### LSD Case and Population Data

This study analyzes the district-level LSD case data from the 2021 and 2022 LSD outbreaks in Uttar Pradesh. LSD data were collected from each district by government veterinarians and were reported to the state level for collation and analysis.

During the 2021 and 2022 outbreaks, real-time case data were collected through active (reported by veterinarians) and passive (reported by farmers to veterinarians) approaches. An LSD case was defined as cattle with clinical signs characteristic of LSD, including generalized skin nodules and enlarged lymph nodes, with or without a confirmatory test.

During the 2021 outbreak, district-level cumulative case data were recorded from the affected districts for the entire outbreak period. The LSD laboratory confirmatory data for the 2021 outbreak were not available. During the 2022 outbreak, district-level cumulative case data were recorded for each affected month (August to November), and laboratory confirmatory tests using polymerase chain reaction (PCR) were conducted on randomly selected nodular/tissue fluid samples (n = 60; positive = 46) from the affected districts (n = 7) to confirm an LSD outbreak. The background cattle population data for each district during the study were obtained from the 20th Livestock Census, 2019 (https://dahd.nic.in, accessed on 5 April 2024).

District-level data on climatic (temperature and humidity) and geographic (land cover) predictors were collected to evaluate their impact on the LSD incidence rate (IR). For climatic factors, the coordinates for each district centroid were used to obtain monthly data on the temperature (measured in Celsius) and relative humidity (expressed as a percentage) for 2022 from the NASA Prediction of Worldwide Energy Resource (POWER) [19], and the average of 4 months of active outbreak (August–November) was calculated for spatial statistical analysis. The district-level land cover data were obtained from Statistical Diary Uttar Pradesh, 2021, by the Economic and Statistics Division State Planning Institute Planning Department, Uttar Pradesh (http://updes.up.nic.in, accessed on 1 April 2024). The land cover was divided into forest area, unculturable land, pasture (and other with trees and plants), fallow (currently unsown land), agricultural (sown) land, culturable wasteland, and non-agricultural land.

### 2.3. Data Analysis

#### 2.3.1. Spatial Analysis

All spatial analyses and map visualizations were conducted in ArcGIS Pro 10.7.1 (Environmental Systems Research Institute, Inc. (ESRI), Redlands, CA, USA). The district-level shape file for Uttar Pradesh was obtained from a publicly available database [20]. Before spatial analysis, the shape file was projected to the Projected Coordinate System UTM/Asia/Indian/1975 UTM/Zone 47N using ArcGIS Pro.

**Figure 2:**
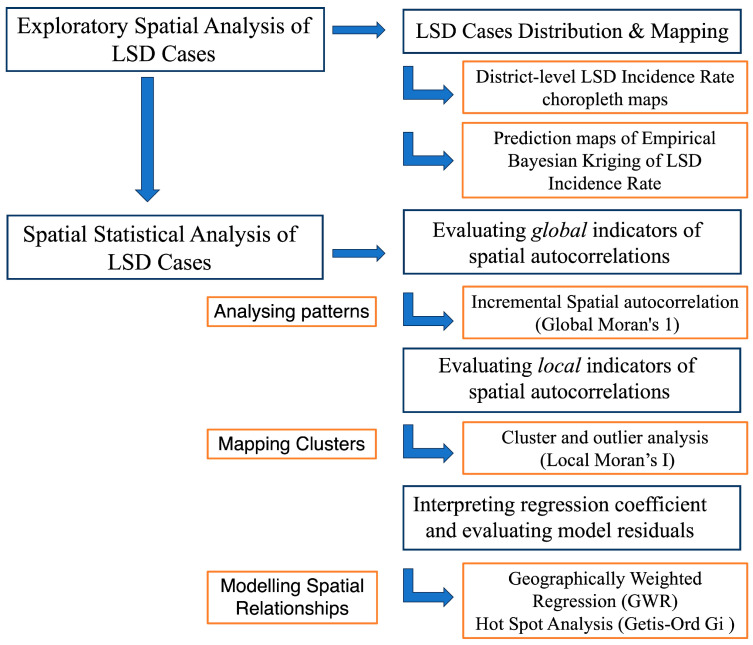
Stepwise spatial analytical framework of the study.

A stepwise analytical framework was used to evaluate and compare the district-level yearly and monthly spatial distribution and heterogeneity of LSD incidence rates (Figure 2).

#### Disease Mapping

The LSD incidence rates per 100,000 cattle (IRs) were calculated for each district in Uttar Pradesh by dividing the total number of LSD cases in that district in a given period by the total cattle population estimates and multiplying it by 100,000. Choropleth disease maps were developed to illustrate IRs by years (2021 and 2022) and months for the 2022 outbreak. While developing the choropleths maps, to minimize within-class variance and maximize between-class variance, the natural breaks (Jenks) classification method was used [21]. A manual adjustment of adding a zero IR category to the Jenks classification was made to highlight the districts with no LSD cases.

The empirical Bayesian kriging (EBK) method [22] was used to interpolate the district-level LSD IRs. The EBK method used semi-variograms to model the spatial variability, quantified the change in LSD IRs by comparing distance and direction [23], and provided estimated LSD IRs for the state of Uttar Pradesh. Isopleth maps using the EBK interpolation of LSD IRs were developed for each year (2021 and 2022) and each month for the 2022 outbreak.

#### Spatial Statistics

For the spatial statistical analysis, every district was represented by a polygon, with its centroid weighted by the LSD IR. The null hypothesis for the spatial autocorrelation analysis assumed that district-level LSD IRs were randomly dispersed, indicating complete spatial randomness. Rejecting the null hypothesis signified that the LSD IRs were spatially clustered or dispersed [24,25].

To assess the global clustering of LSD IRs, the Incremental Spatial Autocorrelation (Global Moran’s I) tool was used with five incremental distance bands. Euclidean distance bands were used to measure distances from each district centroid to its neighboring district centroids. The starting distance assumed that each district has at least one neighbor.

For the conceptualization method, a modified form of a distance decay parameter called the zone of indifference was applied [24,26]. This parameter accounts for zoning and edge effects [26], giving maximum weight to districts within a defined distance band, and beyond that point, the weight decreases as the distance increases. The summary statistics for the global spatial autocorrelation included a Moran’s I Index value, a z-score, and a *p*-value for each distance increment. District-level clustering of LSD IRs was implied by a statistically significant positive z-score > 1.96 and a positive Moran’s I Index [25]. The distance band with the highest z-score (denoted by the maximum peak) in the global cluster analysis was used as the distance band in the local cluster analysis [24,26]. The results of the global cluster analysis of LSD IRs are presented in graphs.

The local spatial LSD IR clusters were detected using the Cluster and Outlier Analysis tool (Anselin local Moran’s I) [24]. The local Moran’s I statistics detected district-level local LSD IR spatial clusters [24,25]. The local Moran’s I statistic identifies high-high areas (districts with a high LSD IR surrounded by districts with a high LSD IR), low-low areas (districts with a low LSD IR surrounded by districts with a low LSD IR), and outlier areas (districts with a high LSD IR surrounded by districts with a low LSD IR or vice versa). A positive local Moran’s I Index with a *p*-value < 0.05 indicated that the target district was surrounded by districts with identical LSD IRs (hot spots/cold spots), whereas a negative local Moran’s I Index with a *p*-value < 0.05 signified that the target district was surrounded by districts with dissimilar LSD IRs (outliers). The results of the local cluster analysis are presented in maps.

Additionally, to model the district-level impact of climatic and land cover determinants on LSD IRs, we conducted an exploratory regression followed by a geographically weighted regression (GWR). These analyses were conducted only for the 2022 outbreak, as detailed information was not available for the 2021 outbreak. For the exploratory regression analysis, the model included LSD incidence as the outcome variable and climatic (temperature and humidity) and land cover (forest, barren, wasteland, pasture, fallow land, sown area, non-agricultural land) determinants as predictor variables. From the exploratory regression analysis, a final model was selected in which the predictor variables explained the most variability in the outcome variable (highest adjusted R^2^, lowest Akaike’s information criterion (AICc), lowest variance inflation factor (VIF), and predictors significant at *p* ≤ 0.01 level). Next, the GWR analysis, using a Poisson model, was conducted to model the associations between LSD IRs in each district as the outcome and temperature, humidity, non-agricultural land, pasture, and fallow land as the predictors [27]. The GWR model allowed the estimation of regression parameters at the local level, overcoming the assumption of spatial stationarity and allowing the relationships between the independent and dependent variables to vary by location (district) [28]. In the GWR model, for local weight assignment and bandwidth calculation, the number of nearest neighbors criterion was employed using the golden search method. To assess the predictive ability of the GWR model, the model residuals were illustrated in a map, and a hot spot analysis (Getis-ord Gi*) of the residuals was conducted to assess the local spatial autocorrelation of the residuals [29,30]. This allowed the detection of local clusters where the model’s performance deviated significantly from expectations. The Getis-Ord Gi* statistic compared the local sum of deviance residuals (i.e., the sum of the residuals of a target area and its neighboring areas) to the overall sum of deviance residuals across the entire study area. This comparison identified areas with significant clustering of high or low residuals. A statistically significant, large positive/negative Z-score indicated a high-rate (hot spot)/low-rate (cold spot) cluster, suggesting that the GWR model underpredicted/overpredicted the LSD IR in these regions, respectively [30,31].

## 3. Results

### 3.1. Descriptive Analysis

Table 1 shows the summary of LSD cases during the 2021 and 2022 outbreaks in Uttar Pradesh. For 2021, only cumulative outbreak data were recorded.

The 2022 LSD outbreak began in August and lasted until November 2022. There were no reported cases in any other months in 2022.

For the 2022 outbreak, the total number of LSD cases and affected districts were almost 20 and 5 times greater, respectively, compared to the 2021 outbreak.

### 3.2. Disease Mapping

**Figure 3:**
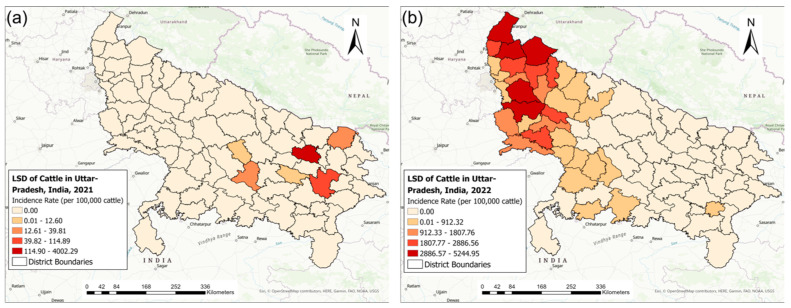
The district-level incidence rates of lumpy skin disease (LSD) (cases per 100,000 cattle) in Uttar Pradesh, India, during the (**a**) 2021 and (**b**) 2022 outbreaks.

Figure 3 illustrates the district-level distribution of LSD IRs (LSD cases per 100,000 cattle) for the 2021 and 2022 outbreaks. The LSD IRs ranged between 5.84 and 4002.29 for the 2021 outbreak and between 0.78 and 5244.95 for the 2022 outbreak (Figure 3). The districts affected by the 2021 outbreak had no LSD cases during the 2022 outbreak (Figure 3).

**Figure 4:**
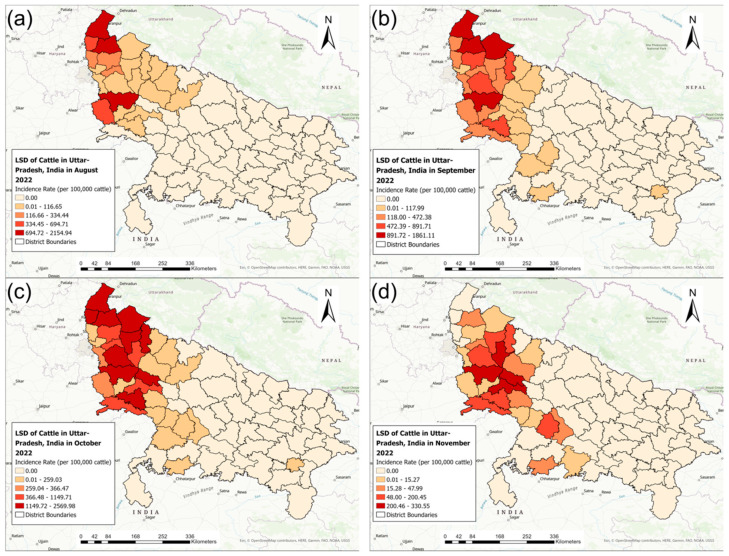
The district-level monthly incidence rates of lumpy skin disease (cases per 100,000 cattle) in Uttar Pradesh, India, during the 2022 outbreak: (**a**) August, (**b**) September, (**c**) October, and (**d**) November.

Figure 4 shows the monthly district-level distribution of LSD IRs during the 2022 outbreak. The biggest district-level LSD IR range was observed during October 2022 (10.60–2569.98), which also marked the peak of the 2022 outbreak. The outbreak started in the northwest region of Uttar Pradesh in districts sharing borders with other Indian states and gradually progressed towards the southeast area of Uttar Pradesh (Figure 4).

**Figure 5:**
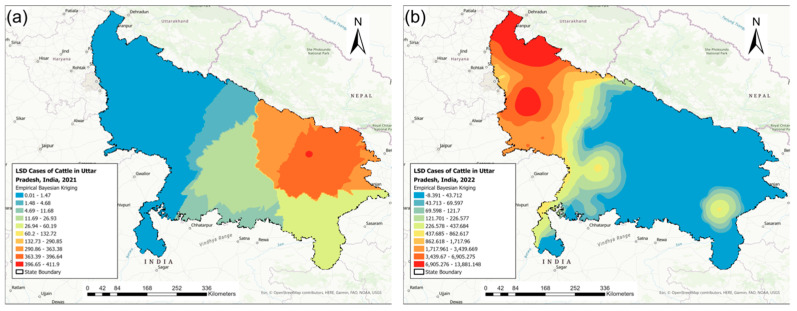
The spatial interpolation of lumpy skin disease incidence rates in Uttar Pradesh, India, using empirical Bayesian kriging: (**a**) 2021 and (**b**) 2022 outbreaks.

The isopleth maps from the EBK model of the LSD IRs detected a high LSD IR region in east Uttar Pradesh during the 2021 outbreak (range from 0.01 to 411.9) and a high LSD IR area in west Uttar Pradesh during the 2022 outbreak (range from −8.391 to 13,881.148) (Figure 5).

**Figure 6:**
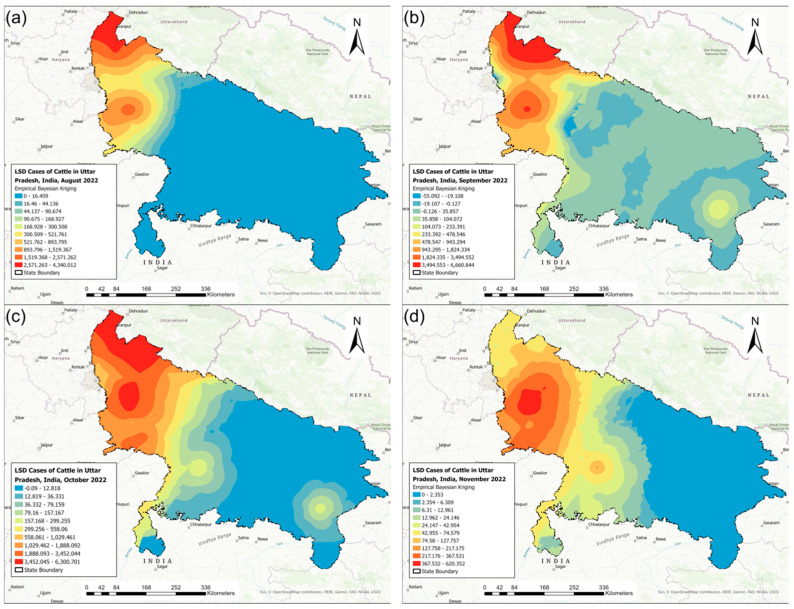
The spatial interpolation of lumpy skin disease incidence rates in Uttar Pradesh, India, using empirical Bayesian kriging illustrated as isopleth maps for each affected month during the 2022 outbreak: (**a**) August, (**b**) September, (**c**) October, and (**d**) November.

Figure 6 illustrates the isopleth maps of the interpolation of LSD IRs for each month during the 2022 outbreak using the EBK method. The map shows the expansion of the high LSD IR region from northwest to southeast Uttar Pradesh between August and November.

### 3.3. Spatial Statistics

The results of the Incremental Spatial Autocorrelation (Global Moran’s I statistics) analysis for the 2021 and 2022 LSD outbreak data showed no significant peak for the LSD IRs in the 2021 outbreak (Supplementary Figure S1a), suggesting the absence of significant global spatial clustering. Therefore, for the local spatial cluster analysis, the default distance of 107.71 km, which ensured that each district had at least one neighbor, was used with the zone of indifference as the conceptualization of spatial relationships.

For the overall 2022 LSD outbreak data, a single peak was observed at a distance of 196.49 km, suggesting maximum spatial global clustering of districts with high LSD IRs at this distance band. Therefore, for the local spatial cluster and outlier analysis of the 2022 LSD outbreak, this distance was used with the zone of indifference as the conceptualization of spatial relationships.

For the monthly Incremental Spatial Autocorrelation analysis of the 2022 outbreak (Supplementary Figure S2), single peaks were identified at a distance of 196.49 km for each month, which were identical to the overall 2022 LSD outbreak analysis (Supplementary Figure S2).

**Figure 7:**
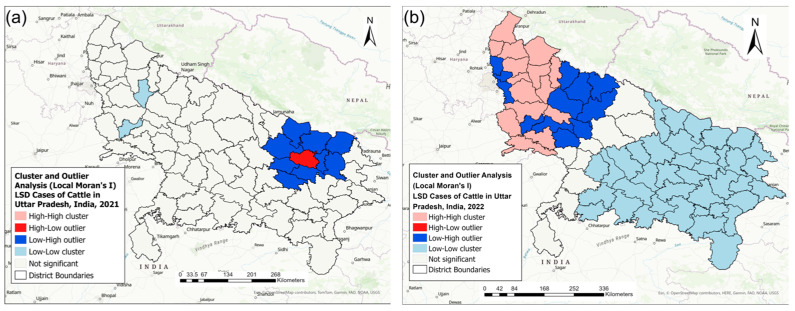
Local spatial clusters of lumpy skin disease (LSD) incidence rates identified by local Moran’s I statistics in the (**a**) 2021 and (**b**) 2022 LSD outbreaks in Uttar Pradesh, India. A Euclidean distance bands of (**a**) 107.71 km and (**b**) 196.49 km were used for the analysis.

The results of the Cluster and Outlier Analysis (Local Moran’s I) of the LSD IRs for each year are presented in Figure 7.

For the 2021 LSD outbreak, one district with high-low and nine districts with low-high LSD IRs emerged in the eastern region of the state, whereas in the western region, two districts with low-low LSD IRs were identified (Figure 7a).

For the 2022 LSD outbreak, 13 districts with high-high and 7 districts with low-high LSD IRs were identified in the western part of the state, and in the eastern part of the state, 44 districts with low-low LSD IRs were identified (Figure 7b).

**Figure 8:**
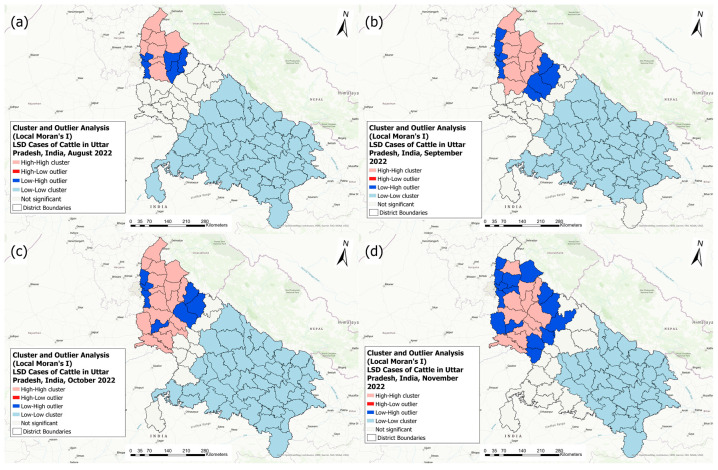
Local spatial clusters of lumpy skin disease incidence rates identified by Moran’s I statistics in each affected month in the 2022 LSD outbreak in Uttar Pradesh, India: (**a**) August, (**b**) September, (**c**) October, and (**d**) November. A Euclidean distance band of 196.49 km was used for the analysis.

Figure 8 presents the local cluster analysis results for each of the affected months during the 2022 outbreak.

In August 2022, 5 districts with high-high, 5 districts with low-high, and 47 districts with low-low LSD IRs were identified (Figure 8a). In September 2022, 10 districts with high-high, 8 districts with low-high, and 44 districts with low-low LSD IRs were identified (Figure 8b). In October 2022, 16 districts with high-high, 7 districts with low-high, and 43 districts with low-low LSD IRs were identified (Figure 8c). In November 2022, 11 districts with high-high, 15 districts with low-high, and 34 districts with low-low LSD IRs were identified (Figure 8d).

The final model from the exploratory regression analysis that was selected included district-level LSD IRs as the outcome and temperature (*p* ≤ 0.05), humidity (*p* ≤ 0.01), pastures (*p* ≤ 0.05), fallow land (*p* ≤ 0.05), and non-agricultural land (*p* ≤ 0.1) as predictors. This model had the following parameters: adjusted R^2^ = 0.27, AICc = 1101.66, and VIF = 1.48.

**Figure 9:**
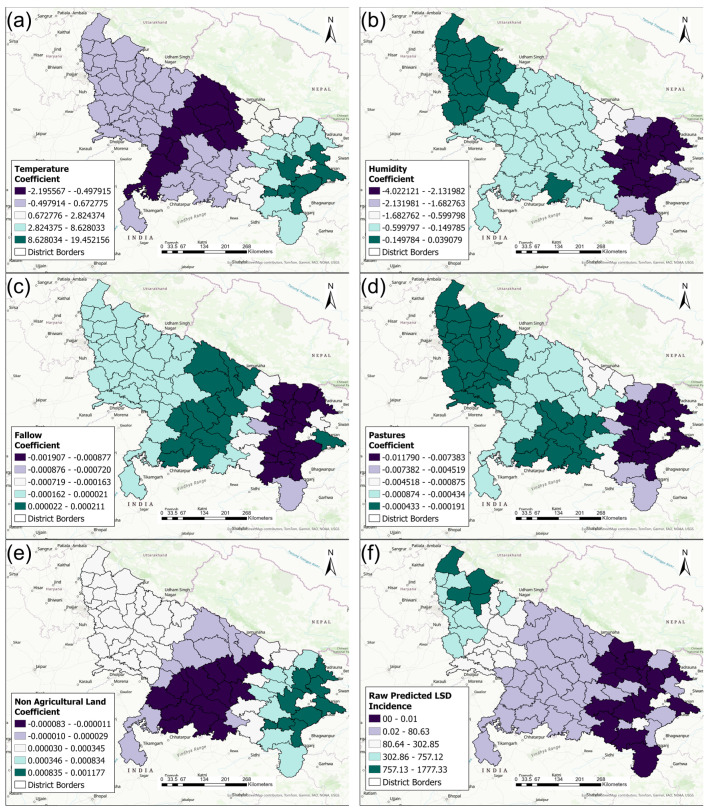
The results of the geographically weighted regression (GWR) Poisson model illustrating the effect of climatic and geographic predictors on the LSD incidence rates in 2022, Uttar Pradesh, India, depicted using model coefficients of (**a**) temperature, (**b**) humidity, (**c**) fallow, (**d**) pasture, (**e**), non-agricultural land, and (**f**) raw predicted LSD incidence.

Figure 9a–e show the local district-level coefficients from the multivariable GWR Poisson model for the relationships between the predictor variables and the outcome (LSD IRs). Temperature (Figure 9a) increased the LSD IRs in the majority of the districts (positive coefficient). Humidity had mainly a negative effect on the LSD IRs, especially in eastern districts (Figure 9b). The land cover variables (fallow land, pasture, and non-agricultural land) showed a weak association and mostly a negative association with LSD IRs (Figure 9c–e).

Figure 9f represents the predicted LSD IRs from the GWR model. The estimated LSD IRs were provided at each location, considering the local influence of the predictors on the LSD IRs. Higher LSD IRs were predicted in the western districts (Figure 9f), which suggests that the model accurately predicted the LSD IRs because this area had the highest observed LSD IRs in 2022 (Figure 3b).

**Figure 10:**
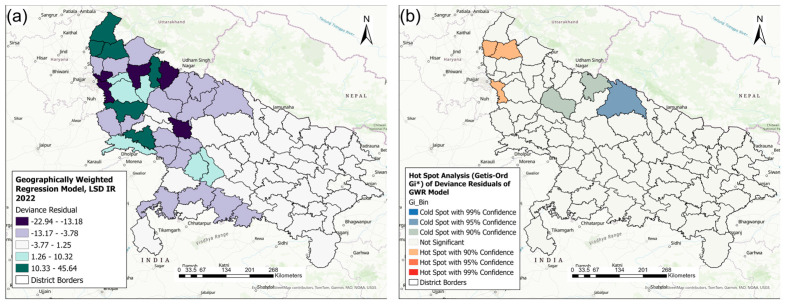
Deviance residuals of the geographically weighted regression (GWR) Poisson model. (**a**) District-level distribution of deviance residuals. (**b**) Hot spot analysis of deviance residuals.

Figure 10 represents the distribution and clustering of the deviance residuals of the GWR model.

The GWR model underpredicted the LSD IRs in 12 districts, where the deviance residuals ranged between 1.26 and 45.64 (Figure 10a), indicating that the observed LSD IRs were higher than the predicted LSD IRs. Of these 12 districts, 6 districts (dark green) had the highest positive deviance residuals, which included states either sharing borders with neighboring states (five districts) or close to state borders (one district) (Saharanpur, Shamli, Muzaffarnagar, Moradabad, Aligarh, and Firozbad) (Figure 10a). On the other hand, the GWR model overpredicted the LSD incidence rate in 23 districts, where the deviance residuals ranged from −3.78 to −22.94. These districts were located in the northwest and southwest of the state.

The hot spot analysis (Getis-ord GI*) of the deviance residuals obtained from the GWR model identified three districts (Shamli, Muzaffarnagar, and Gautam Buddha Nagar) as hot spots (*p* ≤ 0.10) where the model underpredicted the LSD IRs. Additionally, the analysis detected three districts (two districts at *p* ≤ 0.10 and one at *p* ≤ 0.05) as cold spots where the model overpredicted the LSD IRs (Lakhimpur Kheri, Pilibhit, and Budaun) (Figure 10b).

## 4. Discussion

This study assessed the geographical distribution and global and local clustering of LSD IRs at the district level for the 2021 and 2022 outbreaks in Uttar Pradesh, India. A stepwise spatial analytical approach was used to evaluate LSD case data collected by government veterinary officers in each district of Uttar Pradesh, India. The LSD outbreaks were spatially confined, with the 2021 outbreak limited to the eastern part of Uttar Pradesh while the 2022 LSD outbreak involved only the western part of the state. The affected districts in 2021 had no reported cases in the 2022 LSD outbreak, suggesting the role of acquired immunity of the local cattle population. The GWR analysis identified that temperature increased the LSD IRs in the eastern districts, whereas humidity showed a slight positive effect on LSD IRs in the western districts and a negative effect in the eastern districts. Districts with a high proportion of fallow land, pasture, and non-agricultural land had lower LSD IRs.

The 2021 outbreak was restricted to a few districts in the eastern part of Uttar Pradesh, closer to the Nepal border. However, no information on the source of the outbreak was available. A plausible source could be vector-borne transmission or the introduction of infected animals from Nepal, which had an ongoing outbreak in August 2021 [32]. Furthermore, infected animals from other Indian states with LSD-positive status could be a plausible source [33].

For the 2022 LSD outbreak, the largest area with high LSD IRs, illustrated in the choropleth maps, was detected in October. The monthly local cluster analysis of the 2022 LSD outbreak identified several districts with high LSD IRs, and the number of districts with high LSD IRs increased from August to October, indicating the geographical expansion of LSD infections among cattle populations. The distribution of LSD cases during the 2022 outbreak indicates that it initially emerged in districts located in west Uttar Pradesh. The likely source of introduction could be attributed to the unregulated movement of infected cattle or carrier vectors from neighboring states such as Rajasthan, Himachal Pradesh, Haryana, and Uttarakhand, where previous outbreaks had been reported, according to media sources [34,35]. The choropleth maps suggest the progression of LSD inward (to inner districts) and downward (towards southern districts). However, a single eastern district, Varanasi, reported LSD cases during the second month of the outbreak period, with no other nearby district reporting LSD cases after that in the eastern region. This was an interesting finding, and the reported LSD cases in Varanasi could be attributed to unrestricted animal movement, as it was not until mid-September that the Uttar Pradesh government imposed within- and between-state movement restrictions [36]. Additionally, towards the end of August 2022, the Uttar Pradesh state government started vaccinating the susceptible cattle herds using the goat pox vaccine [37], which might provide some immunity as the LSD virus shares almost 96% homology with the goat pox virus, thus enabling the use of the goat pox vaccine for LSD control in the cattle population [38].

The interpolation of the LSD IRs using empirical Bayesian kriging was useful in estimating the local risk of LSD. Spatial interpolation of LSD IRs is a valuable tool that could be used during an ongoing outbreak to anticipate the most likely patterns of disease spread, allowing efficient resource allocation and focused control strategies to high-risk areas [39,40].

The results of the local cluster analysis revealed several districts with high-high LSD IRs in 2022; however, due to limited LSD spread in 2021, no high-high LSD IR district was detected in 2021. The month-wise local cluster analysis of the 2022 LSD outbreak showed an increase in the number of districts with high-high LSD IRs, which peaked in October, followed by a decrease in the number of districts with high-high LSD IRs. Local cluster analysis during an outbreak is useful to identify hot spots (high-high LSD IR areas) where outbreak control methods should focus on preventing the spread of LSD to susceptible areas (low-high LSD IR areas).

The exploratory and GWR analysis results revealed a significant effect of temperature, humidity, and land cover (pastures, fallow land, and non-agricultural land) on LSD incidence rates. Previous studies described the role of climatic (temperature and humidity) and geographic (land cover) factors in influencing the incidence of vector-borne diseases, including LSD, by influencing the vector abundance, their life cycle, and distribution [41]. Our study results showed lower LSD IRs in locations with a higher proportion of fallow and non-agricultural lands. This might be attributable to the abundance and habitat preference of the vector population on these land types. Agricultural lands might be more suitable for vectors due to irrigation and other related agricultural activities that are more likely to promote vector breeding [41,42]. In addition, pastures, during cattle grazing, increase animal interaction and may facilitate LSD transmission. However, in Indian states, including Uttar Pradesh, after 2006–2007, a reduction in pasture areas and the use of these lands for grazing was observed, which might explain the weak negative association in our results [43].

The climatic factors (temperature and humidity) have been shown to have a positive effect on LSD IRs [18,44]. However, our GWR results are inconsistent with the literature, with some districts showing a positive association while others showed a negative association between LSD IRs and temperature/humidity. This discrepancy in the results could be attributed to the non-linear relationship between temperature/humidity and LSD IRs, where extremely high values adversely affect the vector population, reducing LSD incidence. Moreover, for the analysis, average values of temperature and humidity for 4 months (outbreak period) were calculated, masking the subtle nuances in the association. The differences might also be explained by differences in study methodologies, as many previous studies did not use GWR models that account for the local effects of climate on LSD IRs.

The deviance residuals from the GWR and hot spot analyses underpredicted the LSD IRs in some western districts, particularly those close to state borders. This underprediction could be attributed to the non-inclusion of other relevant predictors, such as transport and animal movement across state borders, that may enable LSD transmission [9]. On the contrary, the model overpredicted the LSD IRs in some districts situated away from the western part of the state, where the outbreak was concentrated. Most of these districts did not report any cases or had few cases during the 2022 outbreak. This might be due to the vaccination strategy employed in these districts and limited inter-state animal transport due to distance from state borders.

Considering the study’s limitations, the results presented must be interpreted with caution. The data for this study are subject to reporting bias because during the LSD outbreaks, some cases may have been missed by the field veterinary officers [45]. Moreover, not all LSD cases were confirmed by laboratory tests, and most of the cases were reported based on their clinical signs alone. However, previous studies have used the same approach, as clinical signs of LSD (e.g., skin nodules) are noticeable and testing all animals is not feasible during an LSD outbreak [46,47,48]. Moreover, the data for the 2021 LSD outbreak were provided in a cumulative form for the entire period of the outbreak, providing limited information on the LSD outbreak progression and spread over the outbreak period and not allowing for monthly comparisons. A probable explanation for the lack of detailed case reports could be a lack of instructions on case recording and preparedness among field veterinarians, as 2021 was the first time that an LSD outbreak was detected in Uttar Pradesh. Lastly, multiple LSD virus strains have been reported to cause outbreaks in India, and genetic differences of the virus might influence the emergence and distribution of LSD outbreaks across the study area.

## 5. Conclusions

In conclusion, the geospatial analysis of LSD cases provided insights into the distribution and spread of LSD across districts and described the influence of climatic and land cover factors on the LSD IRs in Uttar Pradesh. Identifying areas with high LSD IRs early into an outbreak might allow priority-based implementation of resources and control protocols, including designing a vaccination strategy. The effect of temperature and humidity on LSD IRs suggests the importance of the vector population in LSD emergence and transmission. Further, districts with a high proportion of non-agricultural and fallow lands and pastures had lower LSD IRs, suggesting the importance of landscape features for vector population suitability. The spatial analytical approaches used in our study could be applied to future LSD outbreaks to help create efficient disease control programs including vaccination strategies, restricting movement to and from highly impacted regions, and directing prevention and control resources to high-risk areas.

## Figures and Tables

**Table 1:** The number of LSD cases and deaths among cattle during the 2021 and 2022 outbreaks in Uttar Pradesh, India.

Year	Number of Districts Affected	LSD Cases	LSD Deaths
2021			
Total	6	5648	Not available
2022			
August 2022	22	18,569	150
September 2022	29	32,871	252
October 2022	32	56,300	226
November 2022	25	4486	47
Total	33	112,226	675

## Data Availability

The data used to support the findings of this study are included in the article.

## Supplementary Materials

The following supporting information can be downloaded at: https://www.mdpi.com/article/10.3390/pathogens13080611/s1, Figure S1. The results of the Incremental Spatial Autocorrelation (Global Moran’s I statistics) analysis of lumpy skin disease (LSD) case data during the (a) 2021 and (b) 2022 LSD outbreaks in Uttar Pradesh, India. Figure S2. The results of the Incremental Spatial Autocorrelation (Global Moran’s I statistics) analysis of lumpy skin disease (LSD) case data for each affected month during the 2022 LSD outbreak in Uttar Pradesh, India: (a) August, (b) September, (c) October, and (d) November.
