# Supplementary material for: Geospatial Analysis of Lumpy Skin Disease Outbreaks among Cattle in Uttar Pradesh, India, 2021–2022
Source: Pathogens. 2024 Jul 24;13(8):611. doi: 10.3390/pathogens13080611 (PMC11357335; doi:10.3390/pathogens13080611)
Supplement: Supplementary file 1 [file pathogens-13-00611-s001.zip › pathogens-3037864-supplementary.pdf]

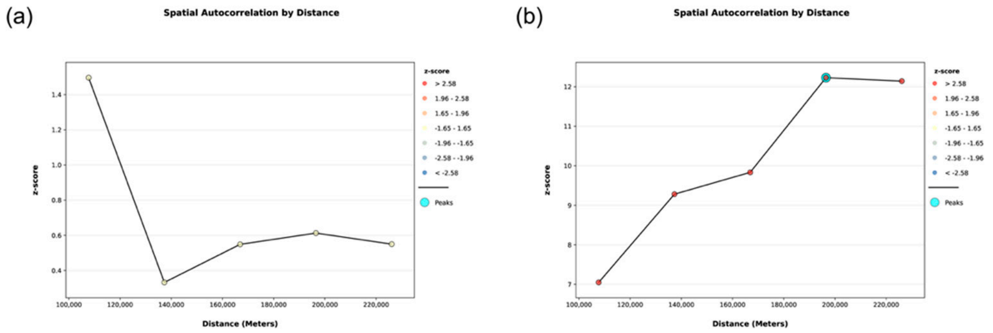

Figure 1. The results of the Incremental Spatial Autocorrelation (Global Moran's I statistics) analysis of Lumpy Skin Disease case data in the (a) 2021 and (b) 2022 LSD outbreaks in Uttar Pradesh, India.

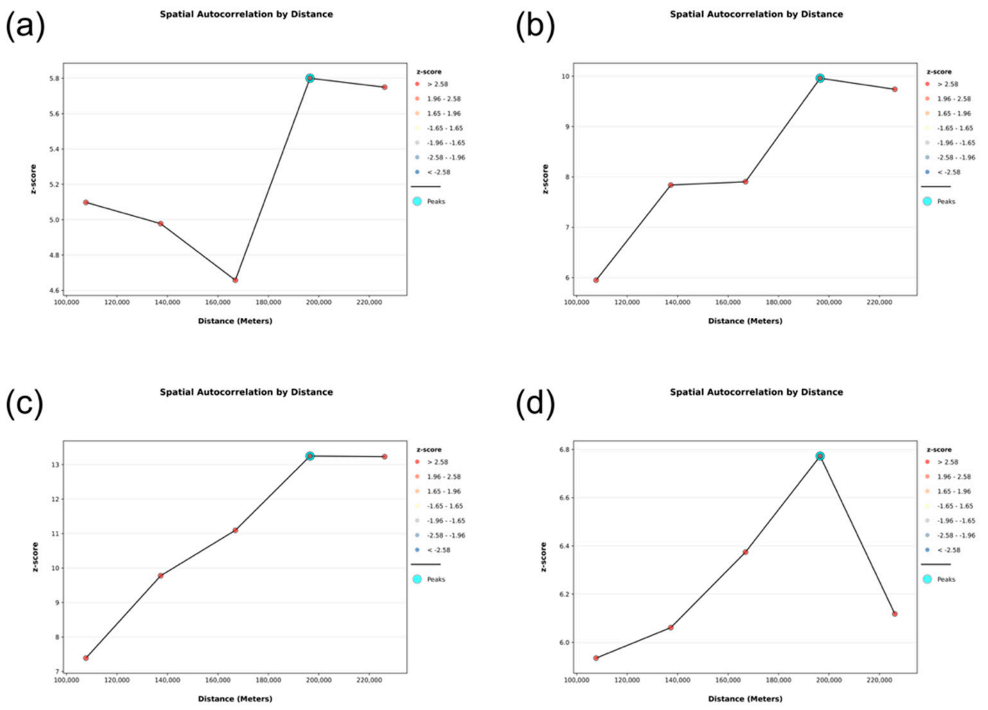

Figure 2. The results of Incremental Spatial Autocorrelation (Global Moran's I statistics) analysis of Lumpy Skin Disease case data for each affected month in the 2022 LSD outbreaks in Uttar Pradesh, India: (a) August, (b) September, (c) October, and (d) November.
